# Benefits of Music Therapy in the Cognitive Impairments of Alzheimer’s-Type Dementia: A Systematic Review

**DOI:** 10.3390/jcm13072042

**Published:** 2024-04-01

**Authors:** María Jiménez-Palomares, Elisa María Garrido-Ardila, Elena Chávez-Bravo, Silvia Teresa Torres-Piles, Blanca González-Sánchez, María Jesús Rodríguez-Mansilla, Álvaro De Toro-García, Juan Rodríguez-Mansilla

**Affiliations:** 1 ADOLOR Research Group, Department of Medical-Surgical Therapy, Medicine Faculty and Health Sciences, University of Extremadura, 06006 Badajoz, Spain; mariajp@unex.es (M.J.-P.); egarridoa@unex.es (E.M.G.-A.); jrodman@unex.es (J.R.-M.); 2 Plena Inclusicion Olivenza, 06100 Olivenza, Spain; elenita1473@gmail.com; 3 Research Group in Immunophysiology, Department of Medical-Surgical Therapy, Faculty of Medicine and Health Sciences, University of Extremadura, 06006 Badajoz, Spain; 4 Department of Nursing, Hospital Don Benito-Villanueva de la Serena, 06400 Don Benito, Spain; mjrmenfesp@gmail.com; 5 Department of Physiotherapy, Jose María Alvarez Health Centre, Extremadura Health System, 06400 Don Benito, Spain; alvaro.detoro@salud-juntaex.es

**Keywords:** music therapy in Alzheimer’s disease, cognitive impairment, occupational therapy

## Abstract

**Background/Objective**: Alzheimer’s disease is a condition that can cause memory, thinking, and behaviour impairments. This type of dementia affects approximately 50 million people globally. Currently, there is no remedy for this disease, but there are different treatment approaches, both pharmacological and non-pharmacological, that try to alleviate the symptoms. The remarkable fact about Alzheimer’s response to music is that musical abilities can be preserved even though language could be lost. The objective of this systematic review is to assess the benefits of music therapy on cognitive impairments in older adults with Alzheimer’s disease. **Methods**: This is a systematic review carried out following the PRISMA guidelines. The literature searches were conducted in the following databases: PubMed, SCOPUS, Cochrane Library, and Dialnet. The inclusion criteria established were as follows: randomised controlled studies and clinical trials published in English and Spanish from 2010 to 2024, patients diagnosed with dementia of the Alzheimer’s type, aged 65 years or older, who had participated in music interventions and had cognitive changes. **Results**: Eleven studies were included in this review. They showed that music therapy interventions mainly improved memory, language, and orientation. The results of a methodological quality analysis showed that six of the articles had good methodological quality and four had excellent methodological quality. **Conclusions**: The results of this review suggest that treatment with music therapy improves cognitive impairments in patients with Alzheimer’s disease. In addition, we can be sure that music creates a link between the patient and the specialist.

## 1. Introduction

Dementia is a group of disorders characterised by a deterioration of the cognitive level, affecting activities of daily living and social functioning. It is one of the leading causes of disability and dependence in older people worldwide. In addition, it has a significant impact not only on those directly affected but also on their caregivers, families, and communities, as well as on society [1].

Around 5–8% of the population over 60 years of age suffers from dementia at some point in their lives. Moreover, it is estimated that by 2030, 82 million people will have some form of dementia [2]. In particular, about 60–70% of the population with dementia suffers from the Alzheimer’s disease type [1].

Alzheimer’s disease is a slowly and progressively degenerative disease that affects memory and mental functioning, but it can also lead to other problems such as confusion, mood swings, and disorientation in time and space. Usually, this condition and its symptoms worsen slowly over time until they are so severe that they interfere with the daily life of the person [3]. It is one of the most disabling and dependence-causing diseases among older people in the world [1].

Old age is considered the main risk factor for developing Alzheimer’s disease. The largest percentage of the population suffering from the disease is 65 years old and over. Ninety per cent of this age group and about one-third of the population over 85 years of age suffer from Alzheimer’s disease [4].

Although there is currently no curative treatment or treatment that can reverse the progression of the disease, numerous interventions are available to improve the lives of both people with dementia and their carers. Non-pharmacological therapies include psychostimulation, cognitive stimulation, reminiscence, art therapy, reality orientation, sensory stimulation, animal therapy, physical exercise, and psychomotor therapy [5]. In addition, music therapy is also included in this type of treatment approach. According to the Word Federation of Music Therapy (WFMT), music therapy is the “professional use of music and its elements (harmony, rhythm, and melody) as a form of intervention in the medical, educational and everyday environment with individuals, groups, families or communities, seeking to optimise their quality of life and improve their health and physical, social, communicative, emotional, intellectual and spiritual well-being”. This approach uses sound, music, movement, and body-sound instruments to develop, produce, and generate a bond or relationship between the music therapist and the patient in order to favour the recovery or rehabilitation process [6].

Human beings have musical abilities of their own, so anyone can benefit from a music therapy process. Even if a person suffers from Alzheimer’s disease, musical abilities are intact. Music therapy interventions in Alzheimer’s disease focus on physical, cognitive, social, and emotional areas [7]. Thanks to the cognitive activation generated through music, all higher functions are stimulated. This enables those functions that are preserved to be stimulated with the objective of lasting as long as possible. Music therapy also works on both verbal and non-verbal communication through the imitation of movements and expressions of gestures. In addition, ideational and ideomotor praxias are stimulated through dance and the use of musical instruments and visual and auditory perception by locating and responding to stimuli, among others [7].

The aim of this review is to assess the benefits of music therapy on cognitive impairments in older people with Alzheimer’s disease.

## 2. Materials and Methods

### 2.1. Study Design and Search Strategy

This systematic review was conducted according to the PRISMA statements and guidelines [8].

A literature review was conducted during the month of February 2024. The search strategy was established in order to analyse the benefits of music therapy on cognitive impairment in older adults with Alzheimer’s disease under study and to update the scientific evidence available in the literature on this subject in recent years.

The following databases were used for the search: PubMed, SCOPUS, Cochrane Library, and Dialnet. The search strategies used were as follows: “cognitive dysfunction”, “Alzheimer”, and “music therapy” translated into Spanish in those databases that required it. The strategies used in each database are shown in Table 1.

The bibliographic references of the studies of interest were manually searched to identify other eligible trials. In line with this approach, four additional studies were included in the analysis.

### 2.2. Eligibility Criteria

The criteria selection was established following the PICO model (population, intervention, control, and outcome). The inclusion criteria were as follows:

- Studies published in Spanish or English.
- Studies published between 2010 and the present (February 2024).
- Clinical trial studies.
- Patients diagnosed with Alzheimer’s disease, aged 65 years or older, who have suffered changes in cognition and have participated in music interventions.

The exclusion criteria were as follows:

- Publications older than 14 years.
- Literature reviews and publications that were not clinical trials.

### 2.3. Study Selection

The pre-selection of the studies was made considering the proposed object of study. Once the search for studies was performed and filters were applied, the researchers reviewed all the titles and abstracts of the identified studies in order to establish their eligibility and inclusion in this systematic review.

The articles that met the inclusion criteria were analysed in detail. The data obtained from the studies included in the review (objective, intervention, duration, variables, assessment tools, and results) were compiled in a table. In addition, the methodological quality of the studies was assessed, and the related data were collected.

All potential full-text articles were independently retrieved and evaluated by two reviewers. The level of agreement between the two reviewers was not specifically calculated. However, any disagreement on the inclusion or exclusion of the full-text articles was discussed and resolved.

### 2.4. Methodological Quality Analysis

The methodological quality of the studies included in this review was assessed with the “PEDro” scale [9]. In 1998, Verhagen developed the PEDro scale to evaluate the methodological quality and possible bias of clinical trials. Although 11 items conform to the scale, the first item is not considered in the total score as it relates to the external validity. The score ranges from 0 to 10 as each item gets a point if the study meets the criteria for methodological quality. The total score is interpreted in such a way that 10 points indicate an excellent methodological quality, scores from 6 to 8 indicate a good methodological quality, scores between 4 and 5 points correlate to a medium quality, and fewer than 4 points show a low methodological quality.

## 3. Results

**Figure 1:**
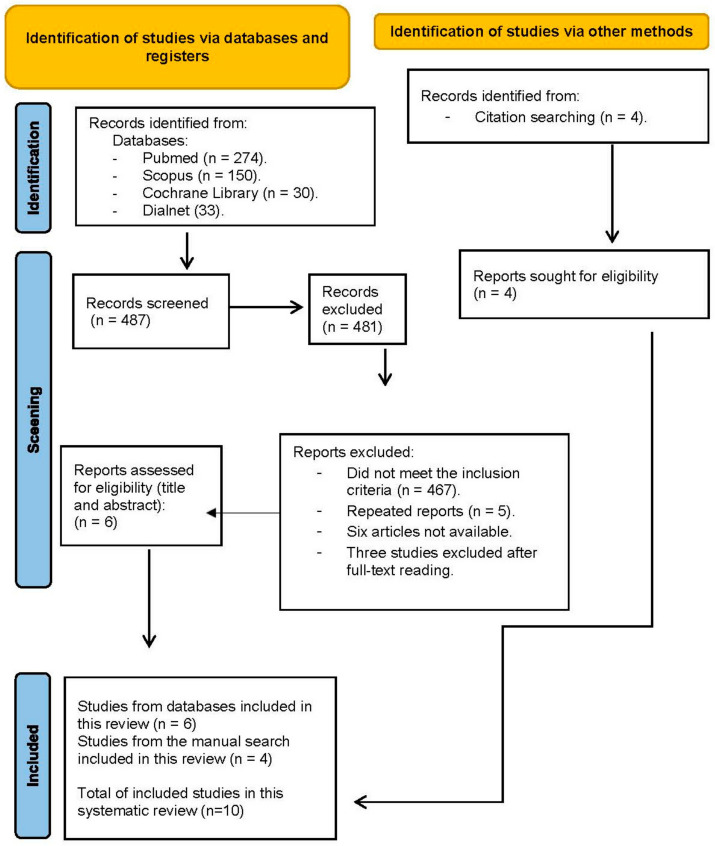
PRISMA flow diagram.

The search in all databases obtained a total of 488 potential studies to be included in this review. Duplicated records and those that did not meet the inclusion criteria were excluded, leaving a total of seven studies included in this review. A total of four articles were obtained from the manual search and included in the review process, leaving a total of 11 studies. The study selection process is shown in the PRISMA flow diagram (Figure 1).

The characteristics of the studies included in this review can be found in Table 2, titled studies included in the review.

### 3.1. Cognitive Function Addressed in the Studies

The clinical trials included in this systematic review aimed to offer a wide range of therapeutic approaches for music therapy in the cognitive approach to Alzheimer’s disease. In particular, two of the included studies focused on the following very specific aspect of cognition: memory. Simmons-Stern et al. [17] aimed to determine whether the benefit of music mnemonics on basic letter recognition in Alzheimer’s disease patients can be extended to content memory. They wrote letters related to common instrumental activities of daily living. In addition, they first selected forty objects related to instrumental activities of daily living together with two actions commonly related to that object. Subsequently, an excerpt from an original nursery rhyme was associated with it, and the lyrics were modified according to the new content to be memorised. In addition, Moltrasio et al. [19] also focused on memory as an object of study, specifically on determining the effect of activating and relaxing music on visual episodic memory in patients with Alzheimer’s dementia. In the experimental group, the participants were presented with 36 images that could evoke different degrees of activation, and after a week, they were given an activating, relaxing, or white noise music treatment. After the music exposure, they had to say which images they remembered, by means of a brief description, followed by a recognition task.

Another study that addressed a very specific area is that conducted by Arroyo-Anlló et al. [18]. Their study focused on the effects of music on self-awareness and the subject’s capacity to understand his/her own states of consciousness. Self-consciousness includes the awareness of one’s own mental states, such as perceptions, attitudes, opinions, and intentions to act.

Gómez Gallego et al. [10,15] and Jihui et al. [13] analysed the effects of music therapy on global cognitive functioning, mental state, and neuropsychological aspects. In addition, one of the articles included in this review, Särkämö et al. [16], focused on caregivers. Their study aimed to assess the efficacy of the use of musical intervention on relatives and carers. Also, two studies comparing the effects of active and receptive music therapy were found in this review, including Särkämö et al. [12] and Gómez Gallego et al. [10,15]. In addition to those authors who attempted to compare the effects of music therapy with respect to other therapies, we can highlight Innes et al. [11], who compared music therapy with a Kirtan Kriya (KK) Meditation programme, Giovagnoli et al. [14], who compared it with a cognitive training programme, and Chéour et al. [20], who were the only ones to compare it with a motor programme, both individually and combined with a music therapy programme.

### 3.2. Music Therapy Techniques

Analysing the most commonly used music therapy techniques, we found that the use of singing was very frequent in experimental groups. A total of four articles in this review used this technique [12,13,16,17] with a repertoire of songs known and selected by the participants or family members as part of their musical preferences. In particular, Sakamoto et al. [12] and Särkämö et al. [16] accompanied this technique with movement, either dancing or clapping and moving. Nonetheless, Jihui et al. [13] only used the singing of familiar songs and Simmons-Stern et al. [17] used children’s songs. Only the study conducted by Giovagnoli et al. [14] used active techniques with a non-verbal approach with free interactions of sound and music using rhythmic and melodic instruments.

On the other hand, we also found studies that used singing, but within a structured music therapy session. In the study by Gómez Gallego and Gómez García [10], each session of the experimental group included several activities as follows: welcome song (patients had to greet and introduce themselves), rhythmic accompaniment activities with clapping and musical instruments (triangles, tambourines, and maracas), movements with background music (several songs were selected and instructions were given to patients to move their upper and lower limbs according to their rhythm and, once a week, dance therapy with hoops and balls was performed), games of recognition of songs and performers (musical bingo was played, and once a week, song recognition was assessed through drawing), and a farewell song (patients were asked to say goodbye). In addition, the second article included in this review, which also used a structured approach, used a welcome song, rhythmic exercises, dance exercises, a music questionnaire, and a farewell song [15].

As for more receptive or passive techniques, the use of music playback was used in experimental groups [11,18,19,20] and control groups [12,15,16]. Arroyo-Anlló et al. [18] and Chéour et al. [20] were the only ones that used music selected by the participants to be listened to. However, Innes et al. [11] and Moltrasio et al. [19] used CDs of music already set by them with instrumental music (Mozart, Bach, Vivaldi, Beethoven, Pachelbel, and Debussy) and classical music (Haydn Joseph’s Symphony No. 70, D major, and Pachelbel’s Canon in D major), respectively.

### 3.3. Treatment Provider and Type of Participants

Regarding the professionals who applied music therapy, we found seven articles in this review in which qualified music therapists [10,12,13,14,15,16,20] conducted the experimental intervention. These studies coincide as they used music therapy with structured sessions [10,15] and with active music therapy techniques such as singing, playing instruments, and rhythmic activities [12,13,14,16]. Studies where the professional applying the music therapy intervention was not specified coincide with those using passive music therapy techniques such as listening to music [11,17,18,19].

All participants of the articles selected for this review were at a mild to moderate stage of dementia [10,11,13,14,15,17,18,19,20], except for those in the study led by Sakamoto et al. [12], who were at a severe stage, and those in the study by Särkämö et al. [16], in which the intervention was performed on caregivers.

### 3.4. Effects of the Intervention

Focusing on the effects on cognitive functions, we found that the application of active music therapy techniques improved cognition in general [10,15,18,20] and showed improvements specifically in verbal fluency [10,13], orientation [10,16], and memory [10,13,16,17,19,20]. It also helped to reduce the behavioural problems of dementia [10,12,15] and improve the level of functionality [15] and mood [12,14,16]. In contrast, more passive activities such as listening to music helped to maintain cognitive functioning [18] and improve emotional and psychological well-being [11].

### 3.5. Methodological Quality Assessment

The scores obtained on the PEDro scale in the analysis of the studies selected for this review are shown in Table 3. After an exhaustive reading of the 11 studies, it could be observed that the scores were in a range with a maximum value of 10 and a minimum of 6 points. The results of the scale showed that six of the articles have a good methodological quality [10,11,12,17,18,19,20] and four have an excellent methodological quality, which are studies [13,14,15,16].

## 4. Discussion

Numerous studies have been carried out with the aim of assessing the efficacy of treatments in older adults with Alzheimer’s disease. However, so far, only a few have been published with the aim of evaluating the benefits of music therapy as a non-pharmacological therapy for older adults with Alzheimer’s disease. In terms of the objective of this review, there are few clinical trials and RCTs that attempted to test the effects of music therapy on cognitive impairments in people with Alzheimer’s disease.

Music therapy is a young scientific discipline with a broad object of study, which has grown from medicine and later also from psychology and musical pedagogy [21]. Therefore, the limitation of this study, which made it difficult to carry out this review, is the insufficiency of scientific articles related to the cognitive benefits of music therapy as a non-pharmacological therapy. This may be related to the slow inclusion of this discipline in the field of health.

In almost all the studies analysed in this review, the sample was very small, so the results from these interventions cannot be generalised to the entire population with Alzheimer’s disease. However, it will help us to continue progressing in the study of this type of non-pharmacological therapy.

Changes in cognition need a certain amount of time to occur [22]. After an exhaustive examination of all the studies included in this review, we observed that most of the interventions have a short duration (approximately 3 months), and few sessions were carried out (an average of 10/12 sessions). Most of the analysed studies obtained significant improvements except for two of them [18,19], whose intervention period was 3 months and two weeks, respectively, and whose conducted sessions were approximately 5 to 7 min. However, one of the studies in which significant improvements were obtained was the study by Jihui et al. [13], which was carried out for six months with two daily sessions of 30–40 min per session, and all the variables studied were improved except for activities of daily living. This suggests that to obtain better results, it would be necessary to apply this type of intervention daily or with longer sessions. This claim is supported by the fact that the studies analysed that did not achieve improvements conducted short sessions during a short period of time.

Regarding the techniques most commonly used in music therapy, we can divide them into passive receptive techniques (the patient’s level of participation is lower) and active or expressive techniques (the patient actively participates in the session) [23]. Music therapy also uses techniques related to sound and body, and the techniques can be divided into the following [24]:

Musical improvisation: Musical improvisation allows the patient to explore aspects of him/herself and aspects in relation to others. It can be vocal, instrumental and/or corporal. It allows the patient to get to know his inner world and to relate to his environment.

Music and movement: Music and movement allow the patient to express himself through his body, connecting his inner world with his environment.

Song: Singing is the union of musical, physical, and social activity, giving rise to a social, motivational, and playful activity.

Musical listening: Musical listening is a technique that facilitates the expression of emotions, concentration, and memory.

Rhythm and percussion: Rhythm and percussion tone and modulate the internal rhythms of the body and improve coordination, motivation, interpersonal relationships…

With regard to techniques, two of the studies analysed in this review used active techniques of music therapy (singing, playing instruments, clapping…) [14,17] and two others were based on passive music listening [11,18,19,20]. In addition, the two types of techniques were combined in the same session in six studies [10,12,13,15,16]. This aspect leads us to believe that this last approach is the best treatment option and the one most frequently chosen in the available research on this subject. The studies included that used the approach with mixed techniques obtained better results in the variables studied, the most favoured being orientation and memory and, to a lesser extent, language.

The use of familiar music makes users relate to their sound identity. Although one of the included studies [13] that used this type of music did not show significant results in cognitive or language function, there was evidence of the positive effect on immediate attention span, delayed word recall and self-awareness, socialisation, and cognition [18]. Music can stimulate emotions, and listening to music has been related to limbic and paralimbic area activation [25]. The use of familiar music in the development of interventions may be one of the main factors in stimulating memories from certain moments in patients’ lives.

Adapting songs that are familiar to patients can help Alzheimer’s patients in advanced stages to talk more, as practising music improves listening and language skills and also helps to promote memories [26]. In the studies analysed in our review, this technique was frequently used [12,16,17,20], showing positive effects on the emotional state of patients [12] and function cognitive globally [20]. Singing has also been found to have a temporary and short-term benefit on working memory [16] and to reduce false recognition and eliminate the mnemonic benefit of music [17].

Visuo-spatial supports facilitate the understanding of people with difficulties in oral communication [27]. In the studies by Giovagnoli et al. [14] and Moltrasio et al. [19], they used verbal support in addition to visuospatial support. In the first of these studies, the results showed that thanks to these supports, social relations improved. Nevertheless, the intervention of neuroeducation and active music therapy and the intervention of cognitive training helped the stabilisation of memory [14]. In contrast, the study by Moltrasio et al. [19] did not obtain significant results. This may be because of the small sample size (n = 28) and the short duration of the study (2 weeks).

Meditation can be useful in a clinical setting as it is associated with states of physiological relaxation that can be used to relieve stress, anxiety, and other physical symptoms, as well as being related to producing cognitive changes [28]. Specifically, in this review we found that in the study carried out by Innes et al. [11], where a Kirtan Kriya Meditation and a music listening programme were completed, perceived stress was reduced, improving the frequency of forgetfulness and the subjective long-term memory function at 26 weeks. These techniques can be incorporated into those already used in music therapy for Alzheimer’s disease patients.

Only one study that combined music therapy with some kind of motor programme was found in this review [20]. The authors compared both programmes separately and combined, obtaining good results in global cognitive function, memory, and functional capacities such as step length, walking speed, and distance covered in the Six-Minute Walk Test. The authors advocated the combined use of both interventions rather than the individualised use of both, which opens up a range of possibilities for music therapy in the motor area.

Regarding the methodological quality of the studies, it can be highlighted that six of the articles have a good methodological quality [10,11,12,17,18,19,20] and four have an excellent methodological quality, which are studies [13,14,15,16]. According to the results of the scale (Table 3), all the studies complied with most of the criteria related to the study design and intervention except for the blinding of the therapists and patients, which is because of the nature of the treatment. The use of the PEDro scale ensures the control of possible bias and a better analysis of the studies. Therefore, the good quality of the research analysed in this review supports the interpretation of the results.

Within the field of music therapy, it is necessary to distinguish between “music in medicine” and music therapy. In “music in medicine”, patients can listen to recorded or live music, and the intervention is punctual and does not form part of any therapeutic process. Its objective is to promote public well-being from a playful perspective, and it is carried out by professional musicians, health professionals, etc. In contrast, music therapy actively involves patients, the intervention is part of the therapeutic process, it is performed by qualified music therapists, and music is the means to an end and patient–professional relationships are established [29]. In this sense, it may be considered a limitation of this review that not all the included studies specified the person who carried out the intervention and applied the treatment. Of the 11 articles included in this review, only 7 specified that the professional who was carrying out the experimental intervention was a qualified music therapist [10,12,13,14,15,16,20]. The rest of the studies either did not explain who the practitioner was [11,18,19] or used a non-health professional, such as a musician [17]. However, what was demonstrated in our analysis is that when active techniques are used, which require the involvement of a professional, the figure of the music therapist is explicit in all of them [10,12,13,14,15,16]. The effects of the interventions can be extrapolated to music therapy, as defined by the Word Federation of Music Therapy (WFMT) [6].

Future research needs to take this limitation into account in order to be able to specify, with more evidence, the benefits of music therapy in older adults with Alzheimer’s disease. This requires that the professionals applying the intervention are music therapists and that this is specified in the methodologies of the studies.

## 5. Conclusions

The results of this review suggest that music therapy improves cognitive impairments in patients with Alzheimer’s disease.

Although the number of publications focusing on the use of music therapy for the management of cognitive impairment in Alzheimer’s disease is increasing, there is still a need for further research in this field in order to improve cognitive impairment in people with this condition.

We can affirm that music creates a communication link between the specialist and the patient, which can improve different cognitive symptoms of Alzheimer’s disease. However, it is necessary that this treatment approach is applied by qualified music therapists in order to extrapolate the results of music therapy itself and not to the use of music in the area of medicine.

## Figures and Tables

**Table 1:** Syntax of combined descriptors in the scientific database search.

Key Words	Database	Total	Repeated	Excluded	Total Search
“cognitive dysfunction” AND “Alzheimer”	PUBMED	12	0	12	0
	SCOPUS	12	0	12	0
	COCHRANE LIBRARY	1	0	1	0
	DIALNET	17	0	17	0
“music therapy” AND “Alzheimer”	PUBMED	6	0	3	3
	SCOPUS	95	0	93	2
	COCHRANE LIBRARY	2	0	2	0
	DIALNET	13	0	12	1
“cognitive dysfunction” AND “music therapy”	PUBMED	251	0	251	0
	SCOPUS	38	0	37	1
	COCHRANE LIBRARY	21	0	21	0
	DIALNET	3	0	3	0
“cognitive dysfunction” AND “Alzheimer” AND “music therapy”	PUBMED	5	0	5	0
	SCOPUS	6	2	6	0
	COCHRANE LIBRARY	6	0	6	0
	DIALNET	0	0	0	0

**Table 2:** Studies included in the review.

Authors	Objective	Intervention	Duration	Variables	Assessement Tools	Results
Gómez Gallego and Gómez García, 2017 [10]	To understand the clinical improvement profile experienced by patients with Alzheimer’s disease.	N = 42 participants Twelve music therapy sessions that included several activities as follows: welcome song, rhythmic accompaniment activities with clapping and musical instruments, movements with background music, song and performer recognition games, and farewell song.	A total of 6 weeks (45 min sessions, twice per week).	Attention, orientation, language, memory, delusions, hallucinations, agitation, anxiety, apathy, irritability, depression, disinhibition, and euphoria.	- Mini-mental state examination. - Neuropsychiatric symptom inventory. - Hospital Anxiety and Depression Scale. - Barthel Index. - HAD Scale. - NPI Scale.	Significant improvements in memory, orientation, depression (*p* > 0.050), and anxiety in mild and moderate patients; in anxiety in mild patients; and in delusions, hallucinations, agitation, irritability (*p* > 0.005), and language disorders (*p* > 0.047) in the group with moderate dementia. The effect on cognitive measures was already noticeable after four sessions of music therapy.
Innes et al., 2016 [11]	To compare the effects of two sessions of Kirtan Kriya Meditation (KK) and music listening (ML) on stress, sleep, mood, and health-related quality of life in older adults with SCD.	N = 60 patients who were divided in a 1:1 ratio as follows: Kirtan Kriya Meditation Group (KK). Music listening group (ML).	A total of 12 weeks of intervention and a 6-month full study.	Perceived stress Sleep Mood Quality of life Subjective memory function Subjective cognitive performance	- Ten-item Perceived Stress Scale (PSS). - Pittsburgh Sleep Quality Index (PSQI). - Profile of Mood States (POMS). - Psychological Well-Being Scale (PWBS). - MOS Short Form 36 (SF-36). - Memory Functioning Questionnaire (MFQ). - Trail Making Test Parts A and B (TMT). - Wechsler Digit–Symbol Substitution Test (DSST).	Significant improvements in both groups at 12 weeks in psychological well-being and in multiple domains of mood and sleep quality (*p*’s ≤ 0.05). Regarding ML, the subjects in the KK group showed greater improvements in perceived stress, mood, psychological well-being, and quality-of-life mental health (*p*’s ≤ 0.09), which were maintained or improved at 6 months. In addition, both groups showed significant improvement in all variables (*p* < 0.05).
Sakamoto et al., 2013 [12]	To compare the benefits among an interactive music intervention, a passive music intervention, and a no-music control group.	N = 39 participants with severe Alzheimer’s disease. They were divided into the following: No intervention control group, N = 13. Passive music intervention group (passive listening to selected music through a CD player), N = 13. Interactive music intervention group (listened to music and participated in interactive activities), N = 13.	A total of 30 min once a week for 10 weeks (10 sessions in total).	Cognitive function Emotions	- Mini-Mental State Examination. - Autonomic Nerve Index. - Face Scale. - BEHAVE-AD.	Passive and interactive music interventions led to short-term parasympathetic control. The interactive intervention showed better results regarding emotional state. The interactive intervention, compared with the passive music intervention and a no-music control condition, achieved a superior long-term reduction in BPSD (behavioural and psychological symptoms of dementia) (*p* < 0.01).
Jihui et al., 2016 [13]	To assess the benefits of music therapy on the cognitive function and well-being of patients with AD.	N = 298 patients with AD (96 with mild dementia, 100 with moderate dementia, and 95 with severe dementia). Divided into three groups as follows: Singing group, N = 100. Lyric reading group, N = 99. Control group, N = 99.	Twice a day, with one session in the morning and one session in the afternoon for 3 months with a duration of 30–40 min per session. Six-month full study.	Cognitive function Neuropsychiatric and behavioural symptoms Activities of daily living	- Clinical Demential Rating scale (CDR). - Auditory Verbal Learning Test (WHO-UCLAAVLT). - Verbal Semantic Fluency Test. - Neuro Psychiatric Inventory (NPI). - Barthel Index.	Overall, music therapy was more effective (*p* < 0.05) in improving verbal fluency and alleviating psychiatric symptoms and caregiver distress than reading lyrics in patients with AD. Music therapy was effective in improving memory and language ability (*p* < 0.05) in patients with mild AD. Also a reduction in psychiatric symptoms and caregiver distress in patients with moderate or severe AD was found. Nonetheless, there were no changes in the results related to activities of daily living in patients with different severities of the disease.
Giovagnoli et al., 2017 [14]	To compare the effects of cognitive training (CT), active music therapy (AMT), and neuroeducation (NE) in patients with mild to moderate AD.	N = 39 patients randomly assigned into three groups as follows: TC: verbal and visuospatial stimuli, N = 13. AMT consisted of a free interaction of sound and music using rhythmic and melodic instruments, N = 13 NE: individual programmes of nutrition, physical exercise, relaxation, coping, N = 13	Each treatment lasted 3 months. CT and AMT lasted 12 weeks, including two 45-min group sessions per week.	Attention Information processing Executive functions Memory	- WTF (phonemic and semantic cues). - SST (initiative and episodic memory). - RAVLT. - RCPM. - Beck Depression Inventory. - State Trait Anxiety Inventory. - Rubben Social Network Scale.	The significant improvements in initiative were higher after CT (38.46%) than after AMT (23.08%) and NE (0%). At 3-month follow-up, verbal initiative (*p* = 0.02) and episodic memory decreased in all patients. Social relationships and mood improved in all three groups but with more entrenchment after AMT and NE (*p* < 0.001). Combining CT and non-cognitive treatments could imply positive clinical implications with better improvements after AMT or NE. In patients with mild to moderate AD, CT might improve initiative and stabilise memory, while non-cognitive treatments may benefit psychosocial symptoms.
Gómez-Gallego et al., 2021 [15]	To compare the effects of two types of musical interventions and a control group.	N = 90 AD patients from six nursing homes. Each nursing home, defined as a cluster, was randomly assigned to receive either an active music intervention (AMI), a receptive music intervention (RMI), or a control intervention.	Each intervention lasted approximately 45 min and took place twice a week for three months (12 sessions in total).	Cognition Behaviour Activities of daily living Motor function	- Mini-Mental State Examination. - Neuropsychological Inventory (NPI). - Geriatric Depression Scale (GDS). - Barthel Index. - Tinetti Scale (TS).	The AMI showed better improvements in cognition, behaviour, and functional status compared with the receptive music intervention and usual care. The effect size of the AMI regarding cognitive deficits and behavioural symptoms was large (η2 = 0.62 and 0.61, respectively). In contrast, it was small to medium (η^2^ = 0.18) for functional status. The receptive music intervention had a stabilising effect on behavioural symptoms compared with the control group (mean change from baseline ± standard deviation = −0.76 ± 3.66 and 3.35 ± 3.29, respectively). The patients who had improved cognitive deficits (85.7), behavioural symptoms (92.9) and functional status (46.4) in the AMI were more numerous than in both the receptive listening (11.8, 42.9, and 14.3, respectively) and control groups (6.3, 12.2, and 17.1, respectively).
Särkämö et al., 2014 [16]	To determine the effectiveness of a new music intervention based on training caregivers of people with disabilities to sing or listen to music regularly as part of daily care.	Eighty-nine pairs of people with disabilities and carers divided into three groups as follows: Singing training group, N = 30. Music listening training group, N = 29. Usual attention control group, N = 30.	One session per week for 10 weeks. Ten sessions in total.	General cognition Orientation Working memory Verbal learning Delayed memory Verbal and visuospatial skills Attention Executive function	- Cornell-Brown quality-of-life scale. - QOL-AD. - Mini-Mental State Examination. - Wechsler Memory Scale III. - CERAD. - Wechsler Adult Intelligence Scale III. WMS-III. - Boston Naming Test (BNT). - Western Aphasia Battery (WAB). - Trail Making Test (TMT). - Frontal Assessment Battery (FAB).	Singing and listening to music showed improvements in mood, orientation, and remote episodic memory compared with the usual care group. Attention, executive function (*p* = 0.039), and general cognition (*p* = 0.41) also improved. Singing also improved working and short-term memory and the well-being of the carers (*p* = 0.026), while listening to music showed benefits on quality of life.
Simmons-Stern et al., 2012 [17]	To investigate the effects of music on the memory of AD patients by making the content of song lyrics relevant to an older adult’s daily life and examining how music encoding alters several different aspects of episodic memory.	Twelve patients with a diagnosis of probable AD and 17 healthy older adults wrote novel lyrics linked to common instrumental activities of daily living and other functions relevant to daily living.	A single session lasting approximately 1 h and 30 min.	Memory Acknowledgement	- Mini-Mental State Examination. - Montreal Cognitive Assessment (MOCA). - CERAD. - Trails.B. - FAS and CAT. - BNT-15.	A group effect was observed as healthy older adults performed better on the recognition task than AD patients. The condition effect was also observed as Pr was better for the singing condition (M = 0.46, SE = 0.037) than for the speaking condition (M = 0.41, SE = 0.040) between groups. No group-by-condition interaction was found [F (1,22) < 1]. Repeated measures ANOVAs were performed with the group and condition factors separately for hit rates and false alarm rates. The ANOVA for hit rates revealed a main effect of condition [F (1,22) = 24.67, *p* <0.001, partial η^2^ = 0.529]. However, no effect of group [F < 1] and no group by condition interaction [F < 1] was observed. False alarm rates were higher for the spoken condition than for the sung condition, so a condition effect was present.
Arroyo-Anlló et al., 2013 [18]	To study the influence of familiar music on self-consciousness (SC) in patients with Alzheimer’s disease (AD).	Forty patients matched by age, educational level, gender, disease duration, and cognitive status were divided into two groups as follows: Experimental group (18 women and two men) with familiar music stimulation. Control group (19 females and one male) with unknown music stimulation.	Approximately 3 months/36 sessions. Each session lasted 45 to 60 min.	Personal identity Anosognosia Affective state Bodily representation Prospective memory Introspection Moral judgements.	- Hachinski Ischaemic Scale. - Mini-Mental State Examination. - Short frontal assessment test (FAS). - Self-awareness questionnaire.	The total score of the SC questionnaire obtained in the experimental group was similar to the scores in the control group (mean ± standard deviations: 11.79 ± 2.44 versus 11.98 ± 2.1; t = −0.74; *p* > 0.09). The results showed significant pre- and post-intervention differences in different aspects of CS between the two AD groups when comparing them. These aspects included personal identity (*p* = 0.019), affective state (*p* = 0.02), moral judgements (*p* = 0.034), prospective memory (*p* = 0.04), and anosognosia (*p* = 0.042). No significant differences were found between the pre- and post-intervention values in the following aspects of CS between the two DP groups: body representation (*p* > 0.05) and introspection (*p* > 0.05). In contrast, there were significant pre-/post-intervention changes in MMS and FAS test scores (*p* < 0.05) in the AD control group, but not in the AD experimental group. The results showed worse scores for the control AD group in the post-intervention phase (MMS: mean ± SD at baseline: 19.90 ± 2.93 vs. after the intervention phase: 17.35 ± 3.57; FAS: 9.11 ± 2.15 versus 6.93 ± 3.41). In contrast, the experimental AD group did notshow any changes in cognitive performance (MMS: mean ± SD at baseline: 19.30 ± 3.68 versus the after intervention phase: 19.5 ± 3.28; FAS: 9.33 ± 2.37 versus 9.04 ± 3.14).
Moltrasio et al., 2020 [19]	To determine the effect of activating and relaxing music on visual episodic memory in AD patients.	N = 28 patients, divided into two groups as follows. Intervention group, n = 17. Control group, n = 11.	Two weeks of intervention. The first session was based on an assessment and after one week, the intervention was carried out, ending with a series of questions and comparing the results.	Immediate recall Delayed recall	- Yesavage Geriatric Depression Scale. - Mini-Mental State Examination. - Clock Test. - California Verbal Learning Test (CVLT). - Spanish Neuropsychological Battery. - King Complex Figure. - Boston Visual Confrontation Dominance Test (BVDT). - Semantic Verbal Fluency (animals). - Trail Making Tests A and B.	There were no significant differences between the groups in the outcomes including age (t (26) = −0.224, *p* = 0.824) and schooling (t (26) = 1.891, *p* = 0.070). However, significant differences were found between MMSE (t (26) = 6.466, *p* < 0.01) and CDT (t (26) = 4.255, *p* < 0.01). In addition, there was a reduction in false positives in the delayed recognition task (one week later). These decreases suggests that the activating musical condition only modulates the recall of visual stimuli.
Chéour et al. 2023 [20]	Evaluate the effectiveness of music therapy and physical therapy used as a single or combined intervention on the improvement in cognitive and motor functions in AD patients.	N = 28 patients divided into four groups as follows: music therapy group (n = 7); physical therapy group (n = 7); music therapy + physical therapy group (n = 7); and control group (n = 7).	Four-month duration (16 weeks) with three sessions per week lasting 60 min.	Cognitive function Motor function	- Mini Mental State Examination. - Alzheimer’s Disease Assessment Scale-Cognitive (ADAS-Cog). - Six-Minute Walk Test (6MWT). - Berg Balance Scale (BBS). - Bessou locometer.	At the end of the intervention, there was an increase in MMEE and a decrease in ADAS-Cog Total and ADAS-COG subscale memory levels in all three experimental groups compared with the control group (ηp2 = 0.56; *p* < 0.01) (ηp2 = 0.61; *p* < 0.001) (*p* (ηp2 = 0.61; *p* < 0.01)), which was more significant in the music therapy + physical therapy group. At the motor level, the step length, walking speed, and distance in 6 min increased significantly in the three groups. The 6 min WT increased significantly in the three experimental groups compared with the control, which was more significant in the music therapy + physical therapy group.

Note: KK: Kirtan Kriya Meditation; ML: music listening; AD: Alzheimer’s disease; HAD Scale: Hospital Anxiety and Depression Scale; NPI: Neuropsychiatric Inventory–Questionnaire; CT: cognitive training; AMT: active music therapy; NE: neuroeducation; RAVLT: Rey Auditory Verbal Learning Test; RCPM: Raven’s coloured progressive matrices; QOL-AD: Quality of Life—Alzheimer’s Disease Scale; CERAD: Consortium to Establish a Registry for Alzheimer’s Disease; SC: self-consciousness; SCD: subjective cognitive decline; FAS and CAT: verbal fluency; BNT-15: 15-item Boston Naming Test; ADAS-Cog: Alzheimer’s Disease Assessment Scale—Cognitive; 6MWT: Six-Minute Walk Test; BBS: Berg Balance Scale; MMEE: Mini-Mental State Examination.

**Table 3:** Assessment of the methodological quality of the studies included in this review.

Authors	1	2	3	4	5	6	7	8	9	10	11	Score
Gómez Gallego and Gómez García, 2017 [10]	1	1	1	1	0	0	0	1	0	1	1	7/11
Innes et al., 2016 [11]	1	1	1	1	0	0	0	1	0	1	1	7/11
Sakamoto et al., 2013 [12]	1	1	1	1	1	0	0	1	0	1	1	8/11
Jihui et al., 2016 [13]	1	1	1	1	1	1	1	1	0	1	1	10/11
Giovagnoli et al., 2017 [14]	1	1	1	1	1	0	1	1	0	1	1	9/11
Gómez-Gallego et al., 2021 [15]	1	1	1	1	0	1	1	1	0	1	1	9/11
Särkämö et al., 2014 [16]	1	1	1	1	1	0	1	1	0	1	1	9/11
Simmons-Stern et al., 2012 [17]	1	1	1	1	0	0	0	1	0	1	1	7/11
Arroyo-Anlló et al., 2013 [18]	1	0	1	1	0	0	0	1	0	1	1	6/11
Moltrasio et al., 2020 [19]	1	1	0	1	0	0	0	1	0	1	1	6/11
Chéour et al., 2023 [20]	1	1	0	1	0	0	0	1	1	1	1	7/11

Note: Criteria 1. eligibility criteria specified; 2. random assignment; 3. concealed assignment; 4. similar groups at baseline; 5. blinding of all subjects; 6. blinding of all therapists; 7. blinding of all evaluators; 8. follow-up of more than 85% of subjects; 9. intention-to-treat analysis; 10. between-group statistical comparisons; and 11. point measures and measures of variability given for at least one key outcome.

## Data Availability

Data are available upon reasonable request to the authors.
